# Nutrient enrichment alters impacts of *Hydrocotyle vulgaris* invasion on native plant communities

**DOI:** 10.1038/srep39468

**Published:** 2016-12-20

**Authors:** Lin Liu, Han Quan, Bi-Cheng Dong, Xiang-Qi Bu, Lin Li, Fu-De Liu, Guang-Chun Lei, Hong-Li Li

**Affiliations:** 1 School of Nature Conservation, Beijing Forestry University, Beijing 100083, China; 2 School of Environmental Science and Safety Engineering, Tianjin University of Technology, Tianjin 300384, China

## Abstract

Nutrients may affect the invasiveness of alien plants and the invasibility of native plant communities. We performed a greenhouse experiment to investigate the interactive effect of invasion by a clonal herb *Hydrocotyle vulgaris* and nutrient enrichment on biomass and evenness of native plant communities. We established three types of plant communities (*H. vulgaris* alone, native plant communities without or with *H. vulgaris*) under low and high levels of nutrients. Native communities consisted of eight native, terrestrial species of three functional groups, i.e. four grasses, two legumes, and two forbs. Invasion of *H. vulgaris* had no effect on biomass of the native community, the functional groups, or the individual species. High nutrients increased biomass of grasses, but reduced evenness of the community. High nutrients also decreased the competitive effect, and the relative dominance index of *H. vulgaris*. Therefore, high nutrients reduced the competitive ability of *H. vulgaris* and enhanced the resistance of the native community to invasion. The results provide a basis for management strategies to control the invasion and spread of *H. vulgaris* by manipulating resource availability to support native communities.

Biological invasion has become a serious ecological problem^1^. Invasion of plant species can decrease native biodiversity^1^,^2^ and alter community structure^3^, biogeochemical cycles^4^ and ecosystem services^1^. In the past few decades, the number of introduced invasive plant species has increased dramatically^5^, and many of them are clonal plants^6^,^7^,^8^. Nutrient enrichment as a result of anthropogenic landscape modifications has also become widely recognized as a serious threat to biodiversity maintenance and ecosystem functioning^9^,^10^. Although many studies have investigated responses of invasive plants and native plant communities to nutrient enrichment^11^,^12^, impacts of nutrient enrichment on the interaction between invasive plants and native plant communities remains an unresolved issue in invasion biology^13^,^14^,^15^.

The invasibility of native communities depends on biotic factors^16^,^17^. For example, native plants can create spatial heterogeneity in soil nutrients^18^ or act as physical obstacles, blocking the spread of the belowground rhizomes and tubers of invasive clonal plants^19^,^20^. The invasibility of native communities also depends on the functional similarity between the invasive species and the dominant species of the native communities^21^. Native species with ecological characteristics that are similar to those of invasive species tend to resist invasions more strongly than those exhibit distinct characteristics because they have a higher niche overlap and thus a higher demand for the same resources^22^,^23^.

Abiotic factors, such as resource enrichment, can confer invasive species with advantages over native species^24^. Nutrients are an important resource and may therefore affect the invasibility of native plant communities and the invasiveness of exotic species^25^,^26^. Many studies have shown that the invasion success of exotic plants can be enhanced by enrichment of nutrients that are limiting^27^. For example, nitrogen addition enhanced richness and abundance of invasive annual herbs^10^,^11^, promoted the spread of introduced plants into terrestrial habitats^12^, but increased the resistance of resident communities to the invasion by *Bromus tectorum*^28^. Thus, different habitat-dependent pathways mediate interactions between invasion and nutrient enrichment to drive community change^29^.

Nutrients are correlated with primary production of ecosystems at large scales because nitrogen and phosphorus are limiting in most freshwater, marine, and terrestrial ecosystems^26^,^30^. Post-industrial anthropogenic activities have amplified nitrogen and phosphorus cycles by 100% and 400%, respectively^30^, and rates of nutrient deposition have increased dramatically^31^. Higher rates of atmospheric nutrient deposition can enhance plant invasion^32^. Native species have different nutrient-acquisition strategies and are key functional components of vegetation. Changes in functional group representation can disrupt key ecosystem function such as productivity. Nutrient enrichment may favor invasive species over native species and thus change the structure and function of communities. However, the complex associations among nutrient enrichment, plant species invasion, and specific invasion patterns are unclear^33^,^34^.

We conducted a greenhouse experiment to test whether high nutrient availability would promote the invasion of a clonal exotic plant species *Hydrocotyle vulgaris* into a terrestrial plant community. We simulated the invasion of *H. vulgaris* into a community consisting of eight native terrestrial plant species of three functional groups (grasses, legumes, and forbs) under two levels of nutrient availability. Specifically, we addressed four questions. (1) Does nutrient availability affect the invasiveness of *H. vulgaris*? (2) Does increasing nutrient availability decrease the invasibility of the native communities? (3) Does nutrient availability affect performance of the functional groups of the plant community? (4) Does the interaction between the invasive plant species and the native community rely on the nutrient availability?

## Results

### The growth of *H. vulgaris*

**Figure 1:**
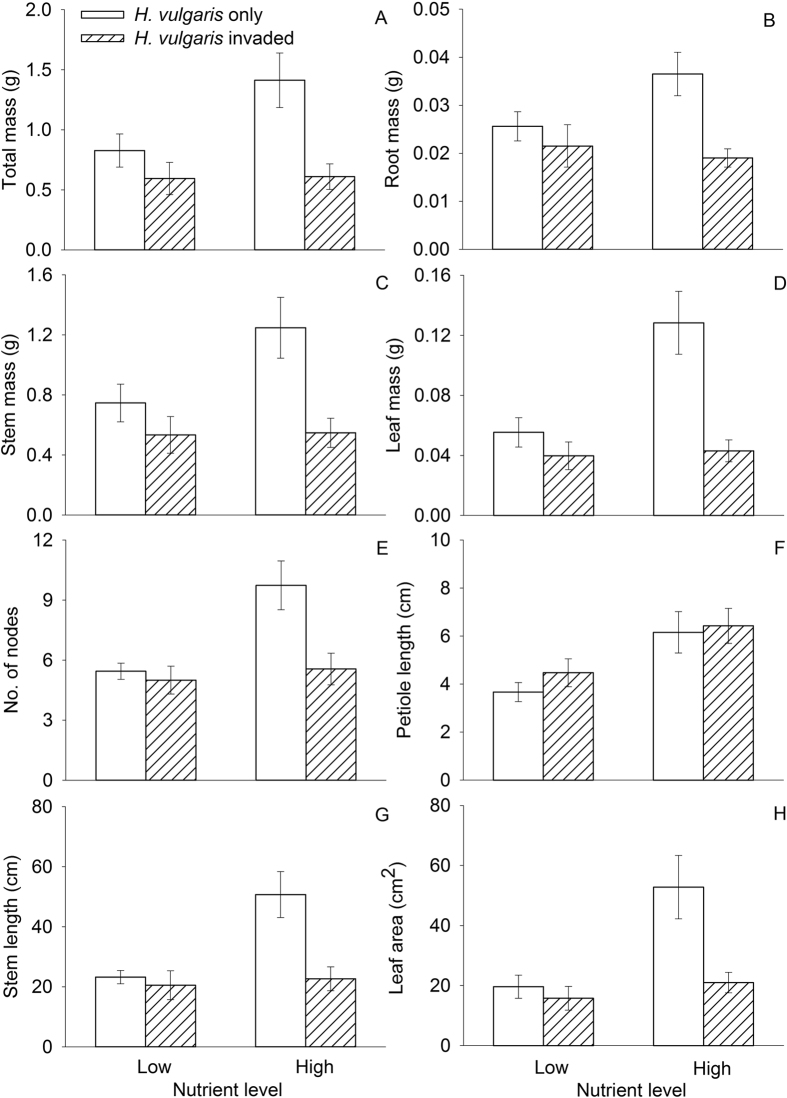
Effects of plant community and nutrient level on the growth of *H. vulgaris* (mean ± SE): (**A**) total mass; (**B**) root mass; (**C**) stem mass; (**D**) leaf mass; (**E**) number of nodes; (**F**) petiole length; (**G**) stem length; and (**H**) leaf area. See Table 1 for ANOVA summaries.

The presence of the native plant community significantly decreased in all traits of *H. vulgaris* measured, except stem mass and petiole length. Nutrient enrichment also increased all traits, except total mass and root mass (Fig. 1, Table 1). There were interactive effects of native plant communities and nutrient enrichment on leaf mass, number of nodes, and stem length of *H. vulgaris*. Nutrient enrichment significantly increased these three traits when *H. vulgaris* grew alone, but had little effect when it grew with the native plant community (Fig. 1D,E and G, Table 1).

### Responses of communities, functional groups, and species

**Figure 2:**
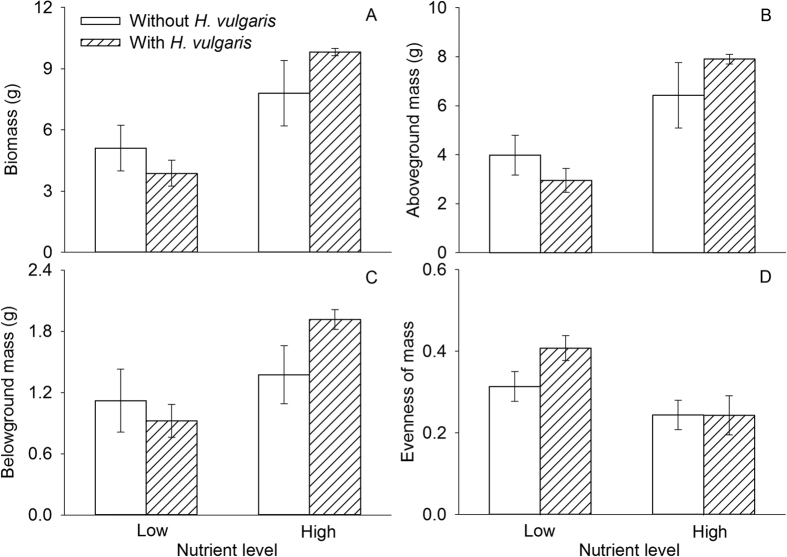
Effects of *H. vulgaris* invasion and nutrient level on measure of the native plant communities (mean ± SE): (**A**) total mass; (**B**) aboveground mass; (**C**) belowground mass; and (**D**) evenness of mass. See Table 2A for ANOVA summaries.

**Figure 3:**
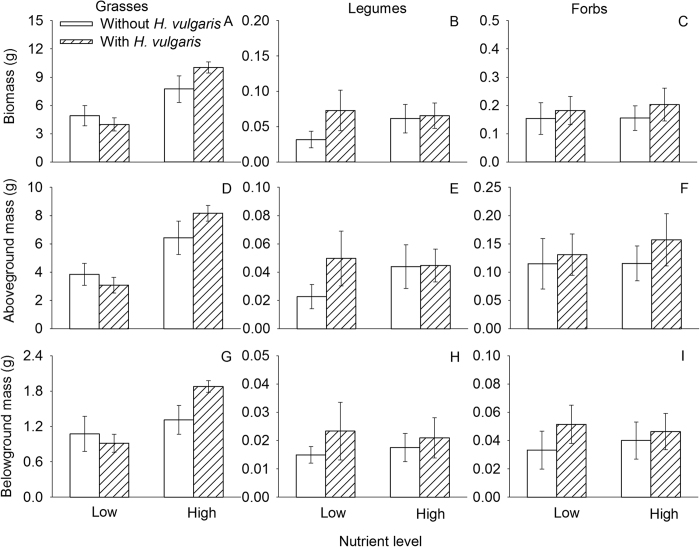
Effects of *H. vulgaris* invasion and nutrient level on biomass of functional groups (mean ± SE). (**A,B** and **C**) total mass; (**D,E** and **F**) aboveground mass; and (**G,H** and **I**) belowground mass. See Table 3 for ANOVA summaries.

Invasion by *H. vulgaris* significantly affected none of the indexes of the native community, but nutrient enrichment significantly increased its biomass and decreased its evenness (Fig. 2A–D, Table 2A). Grasses were the dominant functional group of the native communities and their mass constituted over 90% mass of the native communities (Fig. 3). Invasion by *H. vulgaris* affected none of the parameters of any of the functional groups (Table 3). Nutrient enrichment significantly increased biomass (total, aboveground, and underground) of grasses, but had no effect on that of legumes or forbs.

**Figure 4:**
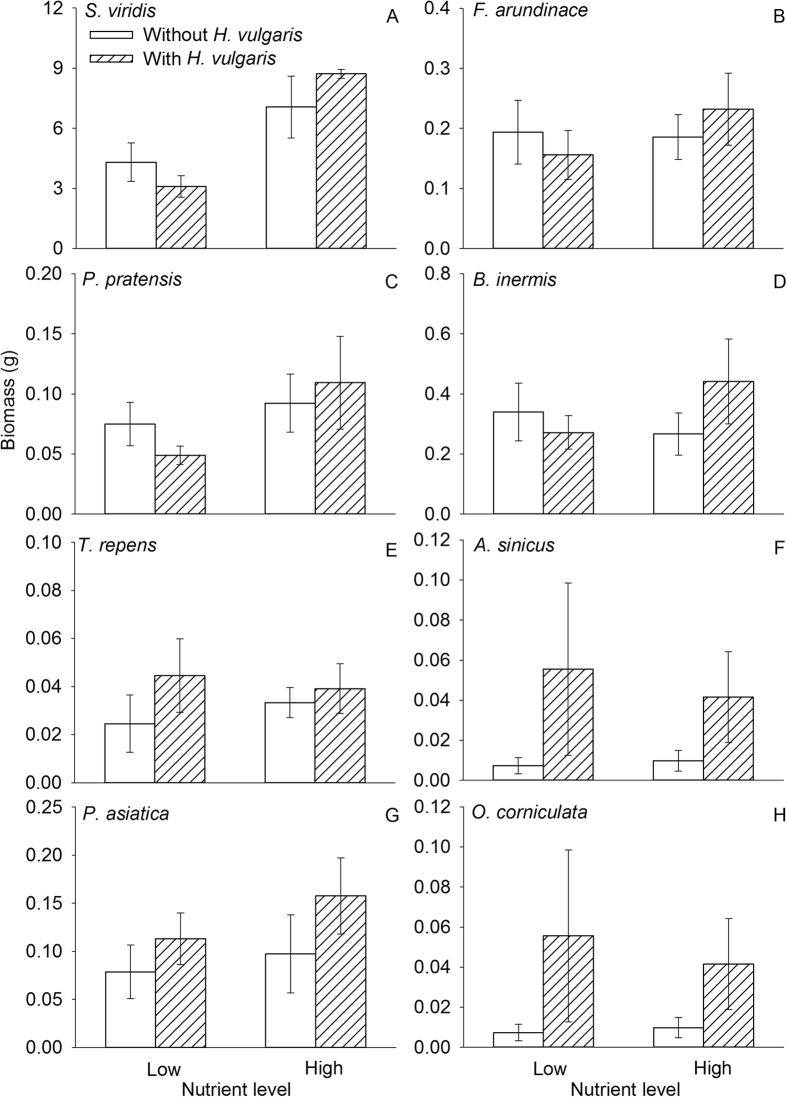
Effects of *H. vulgaris* invasion and nutrient level on total mass of each of eight species (mean ± SE). See Table 2B for ANOVA summaries.

At the species level, *S. viridis* exhibited the highest mass (Fig. 4A). Invasion by *H. vulgaris* had no effect on biomass of any native species, while nutrient enrichment significantly increased biomass of *S. viridis* (Fig. 4, Table 2B). Neither invasion nor nutrient enrichment had a significant effect on seedling establishment of any species, except for *A. sinicus* whose seedling establishment increased due to the presence of *H. vulgaris* (Supplementary Table S1 and Figure S1).

### Interactions between *H. vulgaris* and the native plant community

**Figure 5:**
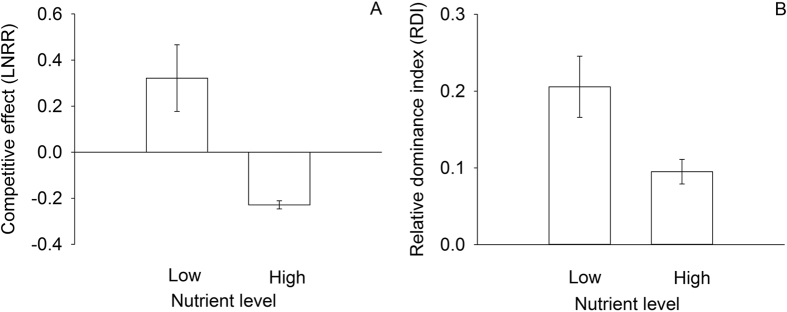
Effects of nutrition level on (**A**) competitive effect and (**B**) relative dominance index of *H. vulgaris* (mean ± SE).

Nutrient enrichment significantly decreased the community-level competitive effect of *H. vulgaris (F*_1,10_ = 14.27, *P* = 0.005) and relative dominance index of *H. vulgaris (F*_1,10_ = 6.61, *P* = 0.027; Fig. 5). Nutrient enrichment can even invert the competitive effect from positive to negative (Fig. 5).

## Discussion

The presence of the terrestrial plant community and nutrient enrichment had opposite effects on the growth and establishment of *H. vulgaris*. There were also significant interaction effects of these two factors on leaf mass, number of nodes, and stem length of *H. vulgaris*. In particular, high nutrient availability enhanced the growth of *H. vulgaris* when growing without a native plant community, but had no effect when growing with the native plant community. Our results thus contradict with those of previous studies, which have typically demonstrated that the invasion success of exotic plants into native plant communities can be enhanced by the enrichment of growth-limiting nutrients^27^,^35^,^36^. There are several potential explanations for this phenomenon. First, biological interactions among species may limit the establishment and spread of introduced species^17^,^37^. The introduced and native species may affect each other by directly competing for soil nutrients, light, water, and physical space^38^. As such, in the high nutrient level treatment, the native species *S. viridis* can capture more light before interspecific competition can suppress its growth, thus developing a higher and denser canopy than *H. vulgaris* and shading *H. vulgaris* and thereby preventing its spread^39^. Alternatively, the spread of stolons and root development at nodes of *H. vulgaris* may have been limited to vacant spaces between native plants, which were diminished by the vigorous growth of the resident root systems under high nutrient conditions^29^. Previous studies have suggested that root phenotypic plasticity within native communities enables native plants to persist in the context of plant invasion or changing resource levels^40^. Furthermore, *S. viridis* can interfere with and suppress the normal root growth of forbs by releasing nonspecific allelochemicals into the rhizosphere^20^. A combination of these factors may explain the observed results.

Overall, *H. vulgaris* invasion had no effect on the recipient native plant community in terms of biomass or seeding establishment for all species except for *A. sinicus*. This result indicates that the resident vegetation community could resist invasion. Nutrient enrichment promoted biomass of the native communities and decreased also their evenness (Fig. 2). The decreased community evenness was because nutrient enrichment mainly increased biomass of *S. viridis* and had no significant effect on biomass of other species. We found no interaction effect of *H. vulgaris* invasion and nutrient availability on biomass of native communities, agreeing with previous findings^29^. However, most previous studies have shown that plant invasion reduces community biomass^41^,^42^,^43^. This discrepancy may reflect differences in characteristics of invasive species or composition of native communities.

We found that nutrient enrichment increased the productivity of the grasses, mainly *S. viridis*, while had little impact on that of the other two functional groups (legumes and forbs). Grasses, especially *S. viridis*, produced the majority of total biomass of the community, suggesting that grasses were the dominant functional group and *S. viridis* was the dominant species in this community. Grasses were more sensitive to nutrient addition and able to obtain more nutrients under the enrichment treatment, as observed in other studies^9^,^10^,^44^,^45^. As such, the resistance of the community to invasion may be attributed to the functional identity of resident competitors. It is likely that the fast-growing, native grasses and the dense canopy formed by them, especially that of *S. viridis*^39^, may have helped the community resist competition from *H. vulgaris*. The absence of interactive effects of these two factors suggests that such differential functional responses to invasion are not dependent on resource availability^46^.

The interaction between the native plant community and *H. vulgaris* was mediated by the nutrient availability. Specifically, nutrient enrichment inverted the competitive effect of *H. vulgaris* from positive to negative, such that *H. vulgaris* only had a detrimental effect on the growth of the plant community when nutrient levels were low. This suggests that changes in nutrient availability altered the competition pattern between the native plant community and *H. vulgaris*, although the influence of *H. vulgaris* invasion was limited. This positive or negative competitive effect suggests that *H. vulgaris* can decrease or increase biomass of the plant community according to nutrient availability. One possible explanation for this facilitation of the community by *H. vulgaris* under high nutrient availability is that exorbitantly high nutrient availability might inhibit plant growth by changing physical and chemical soil properties, causing a nutrient imbalance and reducing photosynthesis by hindering the absorption of Ca^2+^ and Mg^2+^ 47,48. The addition of *H. vulgaris* may have helped to reduce these disruptive nutrient resources and therefore reduce the possible negative effects of high nutrients so that the invaded plant community tended to gain more biomass than the uninvaded community. As shown in a previous study, *S. viridis* grown in close proximity to *Eupatorium adenophorum* accumulates more biomass than *S. viridis* grown in the native control soil^49^. Such impacts may explain the facilitation of the native community by *H. vulgaris* under high nutrient availability.

Moreover, high nutrient availability reduced the relative dominance index of *H. vulgaris* grown in the native plant community. This result suggests that the interspecific competitive ability of *H. vulgaris* was relatively high under low nutrient availability and the ability of the native plant community to resist invasion was relative high under high nutrient availability. Many studies have shown that environmental conditions can change the intensity of interspecific interactions^50^,^51^. Moreover, the interaction between resources and competition may increase the capacity of native plants to resist exotic invasive species by reducing the availability of other resources^28^. Thus, it may be difficult for *H. vulgaris* to spread in a nutrient-rich and species-rich native plant community.

Our results indicate that intrinsic community attributes and nutrient availability can affect the impacts of invasion. Specifically, native species, especially the grass *S. viridis*, may enhance community resistance to invasion by *H. vulgaris*. Accordingly, increases in nutrient deposition due to future global change may not promote *H. vulgaris* invasion into such plant communities, especially those dominated by *S. viridis*. Because our experiment did not test the physiological and biochemical parameters of *H. vulgaris* and the plant community, the potential mechanisms mediating these interactions between *H. vulgaris* and the plant community remain to be explored. Therefore, further studies should be designed to examine the mechanism underlying the interaction between *H. vulgaris* and nutrients available in the rhizosphere to fully understand how nutrient availability affects the invasion process of *H. vulgaris* or other similar clonal plants. In the present study, the effects of *H. vulgaris* invasion on species interactions could not be verified for lack of a monoculture community treatment for each species. To separate the effects on the growth of individual species from effects on competitive interactions among species, it is essential to further construct monocultures containing a single species.

## Methods

### Study species

*Hydrocotyle vulgaris* L. (Apiaceae) is a perennial clonal herb^52^. It commonly occurs in bogs, valleys, and dune grasslands. It was introduced to China as an ornamental plant in the 1990 s and is now considered to be a species with high potential invasiveness^53^. Each ramet, which consists of a leaf and adventitious roots, may be formed by a node along stolons^52^,^54^. In the field, *H. vulgaris* can produce extensive shoot systems and experience heterogeneous micro-environments created by either resource availability or aggregations of neighboring plants^54^. *H. vulgaris* plants used in this experiment were collected from a wetland in the suburbs of Hangzhou, Zhejiang Province, China and were propagated vegetatively in a greenhouse at Forest Science Co. Ltd. of Beijing Forestry University.

The constructed plant communities consisted of a suite of species commonly found in the steppe of northern China^55^. The plant communities consisted of eight species drawn from a random pool. They were classified into three functional groups: four grasses (*Setaria viridis, Festuca arundinacea, Poa pratensis*, and *Bromus inermis*), two legumes (*Trifolium repens* and *Astragalus sinicus*), and two forbs (*Plantago asiatica* and *Oxalis corniculata*).

### Experimental design

We set up three plant arrangement treatments, i.e. *H. vulgaris* alone, a native plant community without *H. vulgaris*, and a native plant community with *H. vulgaris*, crossed with two nutrient addition treatments (i.e., a low or high nutrient level). Each of the six treatment combinations was replicated six times and distributed randomly amongst 36 plastic containers (length, 40 cm; width, 40 cm; depth, 40 cm) filled with 24 L of a 1:1:1 (*v*/*v*/*v*) mixture of quartz sand, vermiculite, and peat. On July 4, 2014, 80 seeds of each of the eight native species were sown uniformly into each container in the treatments with the native plant community with or without *H. vulgaris*. One week later, we planted nine ramets of *H. vulgaris* in the treatment with both the native plant community and *H. vulgaris*, and the treatment with *H. vulgaris* only (no native plant community). Each *H. vulgaris* ramet was composed of a node and a leaf, and was about 10 cm in height.

For the low-nutrient treatment, 100 mL of 0.3 g L^−1^ water-soluble fertilizer (20:20:20, N:P:K; Peters Professional; Everris, Geldermalsen, The Netherlands) was added once every two weeks. The low-nutrient treatment consisted of the application of 1.5 g nitrogen (N) and 1.5 g phosphorous (P) m^−2^ year^−1^. The low-nutrient treatment was designed to simulate filed conditions^56^. In the high-nutrient treatment nutrient concentrations were five times as high as those in the low-nutrient treatment (i.e., 7.5 g N and 7.5 g P m^−2^ year^−1^). The nutrient concentrations in the high nutrient treatment were based on previous research in grasslands^57^.

### Measurements

On September 6, 2014, we harvested the surviving plants of *H. vulgaris* in each container and counted the number of seedlings of each native species. We counted the total number of stem nodes and measured both petiole length and total stem length of *H. vulgaris*. Leaf area of *H. vulgaris* was measured using WinFOLIA (Pro2004a, Regent Instruments, Québec, Canada). All *H. vulgaris* were separated into three parts: leaves, stems, and roots. All plant parts were separately oven-dried at 70 °C for at least 48 hours and then weighed. The surviving plants of each of the forbs in each container were separated into shoots and roots, similarly dried, and weighed. Then, we calculated the sum of the mass, aboveground mass, and belowground mass of all native species as biomass measures of the native plant community. We also calculated biomass of each functional group (grasses, legumes and fobs).

### Data analysis

We calculated the Pielou evenness index as *H*/ln*S*, where *H* is the Shannon–Wiener diversity index based on the proportional final dry mass and *S* is the number of species. *H* was calculated as: *H* = −∑*P*_*i*_ ln (*P*_*i*_) (*i* = 1, 2, …S), where *S* is the number of plant community species and *P*_*i*_is biomass of species *i* divided by the sum of biomass for all eight species in the community^58^.

The competitive effect of *H. vulgaris* was calculated as: competitive effect = ln (biomass of the native community not invaded by *H. vulgaris*/biomass of the native community invaded by *H. vulgaris*)^32^,^59^. A positive value suggests competition between *H. vulgaris* and the native plant community, while a negative value indicates that *H. vulgaris* invasion promotes the growth of the community^59^. We also calculated the dominance of *H. vulgaris* as: the relative dominance index = biomass of *H. vulgaris*/total biomass of all plants in the invaded community^60^.

We performed two-way ANOVAs to test effects of plant community (*H. vulgaris* alone vs. the native community with *H. vulgaris*) and nutrient level (low or high) on total mass, root mass, stem mass, leaf mass, number of node, petiole length, stem length, and leaf area of *H. vulgaris*. We also used two-way ANOVAs to examine effects of invasion (native plant communities without vs. with *H. vulgaris*) and nutrient level (low or high) on total biomass, above- or belowground biomass, evenness of the native plant community, biomass of each functional group, and biomass of each plant species. A one-way ANOVA was performed to test the effect of nutrient level (low or high) on the competitive effect and relative dominance index of *H. vulgaris*.

Data were transformed to natural log or square root before analysis when necessary to remove heteroscedasticity. Leaf mass, stem length, and leaf area of *H. vulgaris* were transformed using the natural log transformation, and petiole length of *H. vulgaris* as well as total mass and aboveground mass of plant communities were transformed using the square root transformation. All data analyses were conducted with SPSS 19.0 (IBM Corp., Armonk, NY, USA).

## Additional Information

**How to cite this article**: Liu, L. *et al*. Nutrient enrichment alters impacts of *Hydrocotyle vulgaris* invasion on native plant communities. *Sci. Rep.* **6**, 39468; doi: 10.1038/srep39468 (2016).

**Publisher's note:** Springer Nature remains neutral with regard to jurisdictional claims in published maps and institutional affiliations.

## Supplementary Material

Supplementary Material

## Figures and Tables

**Table 1:** Summary of ANOVAs for the effects of plant community and nutrient level on the growth of *Hydrocotyle vulgaris.*

Traits	Plant community (P)		Nutrient (N)		P × N	
	*F*_*1,20*_	*P*	*F*_*1,20*_	*P*	*F*_*1,20*_	*P*
Total mass	**10.72**	**0.004**	3.60	0.072	3.25	0.087
Root mass	**8.77**	**0.008**	1.34	0.261	3.37	0.081
Stem mass	3.28	0.085	**10.28**	**0.004**	2.93	0.103
Leaf mass^a^	**14.14**	**0.001**	*7.36*	*0.013*	*4.72*	*0.042*
No. of nodes	*7.73*	*0.012*	**8.49**	**0.009**	*5.05*	*0.036*
Petiole length^b^	0.65	0.429	**11.19**	**0.003**	1.61	0.692
Stem length^a^	**9.42**	**0.006**	*7.66*	*0.012*	*4.69*	*0.043*
Leaf area^a^	*7.66*	*0.012*	**9.55**	**0.006**	3.44	0.079

Values are in bold if *P* < 0.01 and in italics if *P* < 0.05. See Fig. 1 for graphical representation of data.

^a^Indicates log-transformed data.

^b^Indicates square root-transformed data.

**Table 2:** Summary of ANOVAs for the effects of *Hydrocotyle vulgaris* invasion and nutrient level on biomass and evenness of the native communities (A) and biomass of each native species (B).

Traits	Invasion (I)		Nutrient (N)		I × N	
	*F*_*1,20*_	*P*	*F*_*1,20*_	*P*	*F*_*1,20*_	*P*
(A) Native plant communities						
Total mass^b^	0.06	0.808	**17.84**	**0.001**	3.05	0.100
Aboveground mass^b^	0.01	0.942	**20.41**	**<0.001**	3.17	0.094
Underground mass	0.55	0.467	*7.35*	*0.015*	2.58	0.128
Evenness of mass	1.49	0.240	**9.39**	**0.007**	1.53	0.233
(B) Native species						
*Setaria viridis*	0.05	0.819	**19.21**	**<0.001**	2.25	0.152
*Festuca arundinacea*	0.01	0.930	0.48	0.495	0.76	0.397
*Poa pratensis*	0.03	0.859	2.46	0.136	0.75	0.398
*Bromus inermis*	0.31	0.588	0.25	0.623	1.60	0.224
*Trifolium repens*	1.17	0.293	0.02	0.889	0.36	0.556
*Astragalus sinicus*	4.78	0.049	0.10	0.756	0.20	0.661
*Plantago asiatica*	1.89	0.188	0.84	0.374	0.14	0.711
*Oxalis corniculata*	1.12	0.305	0.93	0.350	0.00	0.986

Values are in bold if *P* < 0.01 and in italics if *P* < 0.05. See Figs 2 and 5 for data.

^b^Indicates square root-transformed data.

**Table 3:** Summary of ANOVAs for the effects of *Hydrocotyle vulgaris* invasion and nutrient level on biomass of each functional group.

Traits	Invasion (I)		Nutrient (N)		I × N	
	*F*_*1,20*_	*P*	*F*_*1,20*_	*P*	*F*_*1,20*_	*P*
(A) Grasses						
Total mass	0.54	0.470	**22.82**	**<0.001**	3.01	0.100
Aboveground mass	0.40	0.533	**25.61**	**<0.001**	2.69	0.117
Underground mass	1.10	0.307	**9.63**	**0.006**	3.51	0.077
(B) Legumes						
Total mass	1.13	0.302	0.27	0.609	0.75	0.397
Aboveground mass	0.87	0.364	0.30	0.590	0.78	0.388
Underground mass	0.69	0.416	0.00	0.991	0.12	0.732
(C) Forbs						
Total mass	0.54	0.473	0.05	0.832	0.03	0.856
Aboveground mass	0.52	0.479	0.11	0.741	0.10	0.753
Underground mass	0.86	0.366	0.00	0.953	0.21	0.655

Values are in bold if *P* < 0.01 and in italics if *P* < 0.05. See Fig. 3 for data.
