# Supplementary material for: Nutrient enrichment alters impacts of Hydrocotyle vulgaris invasion on native plant communities
Source: Sci Rep. 2016 Dec 20;6:39468. doi: 10.1038/srep39468 (PMC5172361; doi:10.1038/srep39468)
Supplement: Supplementary Material [file srep39468-s1.pdf]

# Nutrient enrichment alters impacts of *Hydrocotyle vulgaris* invasion on native plant communities

Lin Liu[1], Han Quan[1], Bi-Cheng Dong[1], Xiang-Qi Bu[1], Lin Li[1], Fu-De Liu[2], Guang-Chun Lei[1], Hong-Li Li[1*]

1 School of Nature Conservation, Beijing Forestry University, Beijing 100083, China

2 School of Environmental Science and Safety Engineering, Tianjin University of Technology, Tianjin 300384, China

*corresponding author: Hong-Li Li (lihongli327@163.com)

**Supplementary Table S1.** Summary of ANOVAs for the effects of *H. vulgaris* invasion and nutrient level on No. of seedling establishments among containers for each species.

| | Invasion（I） | | Nutrient level (N) | | I ×N | |
|---|---|---|---|---|---|---|
| | $F_{1,20}$ | $P$ | $F_{1,20}$ | $P$ | $F_{1,20}$ | $P$ |
| *Setaria viridis* | 0.02 | 0.895 | 0.55 | 0.470 | 2.83 | 0.112 |
| *Festuca arundinace* | 0.06 | 0.817 | 1.09 | 0.312 | 0.33 | 0.575 |
| *Poa pratensis* | 0.27 | 0.610 | 0.55 | 0.468 | 0.01 | 0.942 |
| *Bromus inermis* | 0.23 | 0.638 | 0.52 | 0.483 | 0.47 | 0.504 |
| *Trrifolium repens* | 0.25 | 0.625 | 0.17 | 0.683 | 0.34 | 0.569 |
| *Astragalus sinicus* [b] | **9.87** | **0.006** | 0.78 | 0.391 | 0.45 | 0.511 |
| *Plantago asiatica* [b] | 0.67 | 0.427 | 0.05 | 0.828 | 0.06 | 0.804 |
| *Oxalis corniculata* | 3.88 | 0.066 | 0.01 | 0.926 | 0.00 | 1.000 |

Values are in bold if $P < 0.01$ and in italics if $P < 0.05$. See Supplementary Figure S1 for data. [b] indicates square root transformed data.

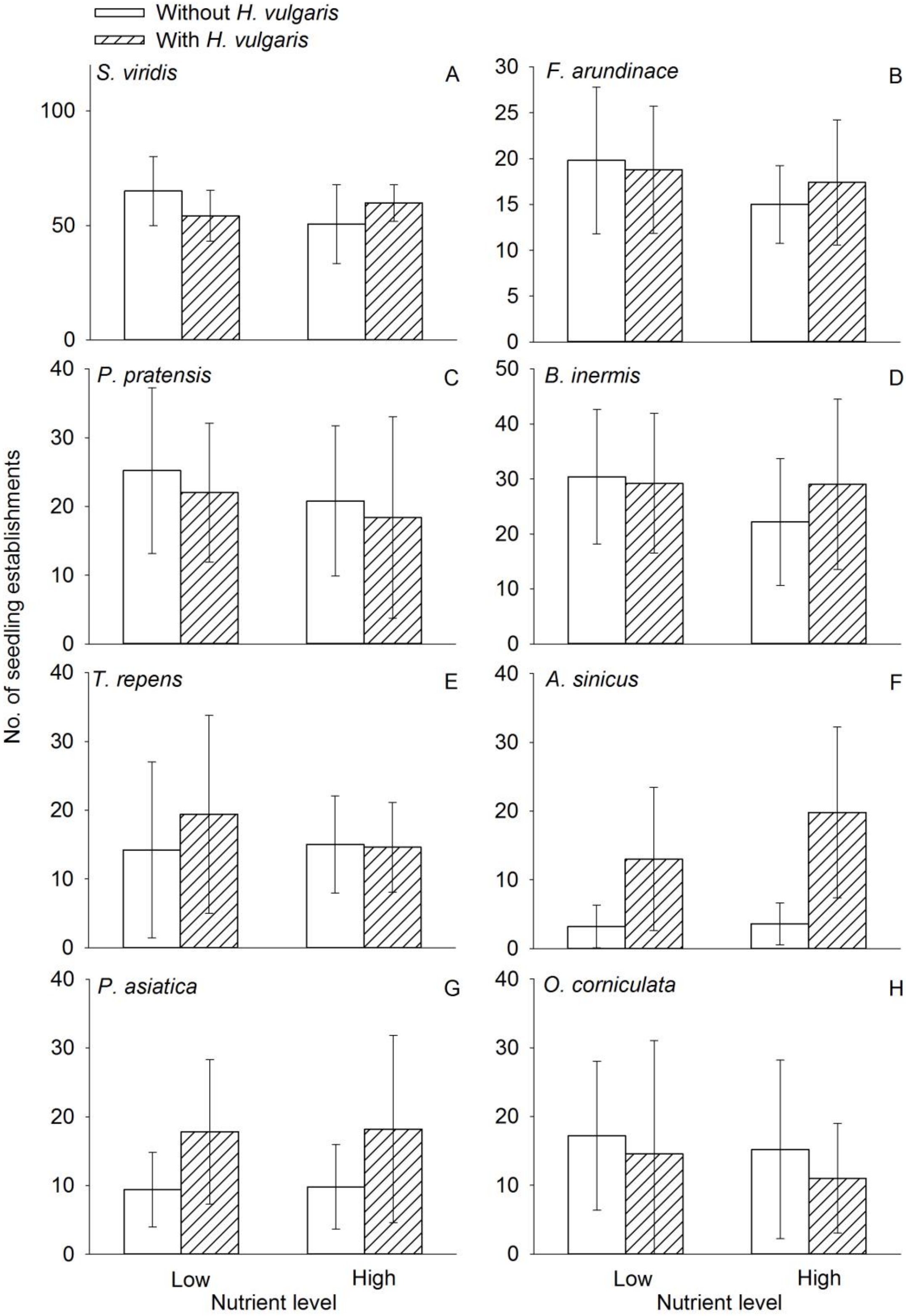


**Supplementary Figure S1.** Effects of *H. vulgaris* invasion and nutrient level on No. of seedling establishments among containers for each species (mean ± SE). See Supplementary Table S1 for ANOVA summaries.
